# Selection of the intrinsic polarization properties of animal optical materials creates enhanced structural reflectivity and camouflage

**DOI:** 10.1098/rstb.2016.0336

**Published:** 2017-05-22

**Authors:** Kathryn D. Feller, Thomas M. Jordan, David Wilby, Nicholas W. Roberts

**Affiliations:** 1 School of Biological Sciences, University of Bristol, Bristol BS8 1TQ, UK; 2 School of Geographical Sciences, University of Bristol, Bristol, BS8 1SS, UK

**Keywords:** structural colour, photonics, Anderson localization, evolution

## Abstract

Many animals use structural coloration to create bright and conspicuous visual signals. Selection of the size and shape of the optical structures animals use defines both the colour and intensity of the light reflected. The material used to create these reflectors is also important; however, animals are restricted to a limited number of materials: commonly chitin, guanine and the protein, reflectin. In this work we highlight that a particular set of material properties can also be under selection in order to increase the optical functionality of structural reflectors. Specifically, polarization properties, such as birefringence (the difference between the refractive indices of a material) and chirality (which relates to molecular asymmetry) are both under selection to create enhanced structural reflectivity. We demonstrate that the structural coloration of the gold beetle *Chrysina resplendens* and silvery reflective sides of the Atlantic herring, *Clupea harengus* are two examples of this phenomenon. Importantly, these polarization properties are not selected to control the polarization of the reflected light as a source of visual information *per se.* Instead, by creating higher levels of reflectivity than are otherwise possible, such internal polarization properties improve intensity-matching camouflage.

This article is part of the themed issue ‘Animal coloration: production, perception, function and application’.

## 1. Introduction

Animals use structural optics to produce highly reflective coloration [1–4]. Many of these optical structures follow well-understood physics [5]; however, there are also several examples where no synthetic analogues exist [6]. Structural optics represents the most efficient solution for creating coloration. The architectures responsible for structural colour can undergo adaptation with little inclusive cost or expense. Only small changes in the initial properties of materials are required for a structural optical mechanism to access any point within the visual colour space of an intended animal receiver. However, the natural world has to rely on a limited diversity of materials to produce structural reflections, typically using chitin [7,8], guanine [1,9] or the regulation of the protein reflectin [10]. In many cases such structural coloration has been under strong selective pressure, whether in the context of sexual selection, for example, the remarkable nape feather displays of the species of birds of paradise [11], or under natural selection where structural colours provide forms of camouflage [9,12] or aposematic signals [2].

This is the standard picture of structural optics, one where the spectral reflectivity is controlled by a combination of the spatial arrangement and isotropic optical properties of the structure [1,5]. However, the intrinsic polarization properties of biological materials [6,13–15] can also strongly influence the task-related function of structural optics. In this work we use the term intrinsic polarization properties to refer to optical properties such as refractive index or birefringence (the difference between two refractive indices); these are properties that are inherent to individual materials but affect the polarization of light. The polarization of light, or just polarization, is a term used to describe three characteristic physical properties of electromagnetic waves. (i) The *angle of polarization* describes the average angle at which the electric fields of the waves of light oscillate, and (ii) the *degree* or *percentage polarization* defines the ratio of the (averaged) intensity of the polarized portion of the beam to its total (averaged) intensity [16]. Polarization can also have a circular component and (iii) the *ellipticity*, which ranges from −1 (left-handed circularly) to 0 (linearly) to 1 (right-handed circularly) polarized light, respectively. It is well understood that many animals, such as insects [17,18], crustaceans [18] and some vertebrates [18–21], exhibit different levels of visual sensitivity to the polarization of light. It is established in many of these cases that visually guided behaviours depend on polarization information found in natural light environments [22,23]. Nonetheless, it is not the polarization of light *per se* or associated behaviour we are concerned with in this paper, and this is an important point to communicate. Our objective is to discuss only the intrinsic polarization properties of the materials and structures, and to establish how these properties can be under selection to control the overall reflectivity in novel ways, something that has never been addressed before.

The often iridescent, and metal-like reflections from the insect order Coleoptera are probably the most widely studied examples of structural colour in nature, dating back to Michelson in the 1920s [24], who first began to examine the optical mechanism responsible. In the 1970s Neville established the more general link that chitinous structures display a close similarity to cholesteric liquid crystal materials in the way they self-assemble to form a helical organization based on the intrinsic chirality of the constituents [25]. This underlying chiral design plan has a significant number of advantages, particularly in terms of ease of spectral manipulation. Relatively simple structural variations can change the optics considerably. Changes to the pitch (helix repeat distance) can move the wavelength of maximum reflection, and a distribution in the pitch can create a broadband reflector [26]. Moreover, spatial variation in the pitch and the creation of a pointillist surface manipulates the spectral signature in the eyes (and visual acuity) of the intended receiver [27–29].

Terrestrial animals are not alone in their use of structural coloration. The silvery reflectors in the sides of many species of fish are one-dimensional, disordered photonic structures consisting of alternating layers of guanine and cytoplasm, analogous to optical structures known as distributed Bragg stacks [9]. These stacks act as mirror-like reflectors and are used by the fish for camouflage in the open ocean [9]. E.J. Denton, J.A.C. Nicol and M.F. Land carried out the vast majority of the original physical characterization of reflective guanine crystal stacks in fish in the 1960s and the early 1970s [9,30–33]. However, more recent investigations into the polarization and disordered optics that underlie these broadband reflections—in particular the effect of the remarkably high optical anisotropy (birefringence) of the guanine crystals—provide a greater understanding of the structure, the optics and the selective pressure to match the background radiance [6,34–37].

It is the symmetry properties of the underwater light field that provide an explanation for how the guanine multilayer reflectors in fish are able to function as an effective camouflage strategy [9,31]. While in the upper layers of the open ocean the radiance distribution of light is strongly dependent upon the position of the sun, with increasing depth the underwater radiance distribution becomes more symmetrical about the vertical axis [38]. At a certain depth this can be approximated as being cylindrically symmetric about the vertical axis [9,39,40]. In this ideal open-ocean light environment, a vertical 100% reflective mirror provides an ideal form of camouflage, perfectly matching the intensity and spectral properties of the background light field (see fig. 6.7 in [41]).

In this paper, we use examples of chiral chitin–based reflectors of the beetle, *C. resplendens*, and the birefringent guanine crystal stacks of fish such as *Clupea harengus* (Atlantic herring) to demonstrate how the intrinsic polarization properties of the optical structures increase the reflectivity over a range of viewing angles. Specifically, for both structures, we demonstrate how the intrinsic polarization properties enable reflectivity values to be increased above a theoretical threshold of approximately 50% that occurs in similar structures that are not affected by the symmetry breaking of the chirality or birefringence. Again, we would like to stress that these properties are not being selected to affect the polarization of the reflected light, but rather to control and improve the overall reflectivity. This is a new idea for considering the evolution of structural coloration.

## 2. The polarization properties of biological reflectors

Many of the optical materials and arrangements of optical materials found in the structural reflectors of animals are anisotropic; that is, exhibit a structural dependence on direction and the polarization state of the light. Optical anisotropy can arise through a variety of mechanisms that are characterized by the length scale relative to the optical wavelength: (i) Intrinsic anisotropy is due to atomic structure, for example intrinsic birefringence is defined as the difference between two refractive indices of a material, or chirality which creates an optical handedness. (ii) Form birefringence is an induced difference in the effective refractive indices of a material due to sub-wavelength periodic structure in that material [42,43]. The effective refractive indices that the incident light experience are defined by the boundary conditions of Maxwell's equations [42]. (iii) Finally, structural anisotropy occurs from anisotropic arrangements of scattering elements at the order of the wavelength and results in different optical responses due to different periodicities (and associated Bragg resonances) in different directions [44]. Table one illustrates the scale hierarchy of anisotropy present in biological reflectors and gives several examples where material anisotropy and chirality, and structural anisotropy affect the optical response.

Intrinsic and form anisotropy, as described above, can readily be included in theoretical calculations of the reflectivity from one-dimensional biological reflectors using the 4 **×** 4 transfer matrix technique [45,46]. While we do not set out the theory again here, it is important to note that this technique represents an exact numerical solution to Maxwell's field equations [47]. Transfer matrix models of biological reflectors typically then apply a statistical averaging procedure when calculating the reflectivity, which accounts for local variation in the reflective structure that averages out when illuminated by a macroscopic light source [35,36]. For off-axis illumination, isotropic one-dimensional models of biological reflectors, and the majority of anisotropic models, are predicted to have a Brewster angle, approximately 50–55 degrees for most isotropic biological materials. At the Brewster angle the polarization component of light that is polarized tangentially to the plane of incidence is completely transmitted, and is an effect that arises purely due to the geometry of the stack system (table 1).

**Table 1.** The intrinsic polarization properties of biological materials that influence the functional optics of the animal reflective structures. The table shows the progression of length scales at which anisotropy is present; from the atomic level and the control of refractive index and birefringence to visible wavelengths, where anisotropic structures such as diffraction greetings control the polarization dependence of scattering and interference.

anisotropy group	references	length scale and structure
*intrinsic anisotropy*—intrinsic birefringence		
Intrinsic anisotropy occurs at the atomic scale due to asymmetric electronic properties, and determines the polarizability and directional dependence of the refractive index.		
guanine crystals—fish	[1,6,48–50]	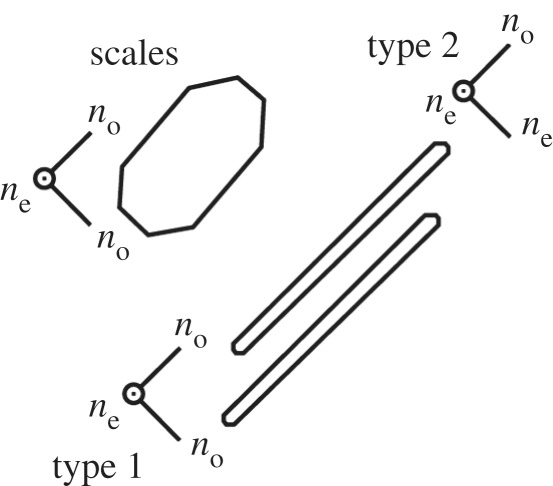
guanine crystals—spiders The guanine crystals in fish and spiders are approximately uniaxial crystals with refractive indices of *n*_o_ = 1.83, *n*_e_ = 1.46. Typically the high, *n*_o_, refractive index values are orientated in the broad planes of the crystal (Type 1), but sometimes the *n*_e_ value is orientated in the broad plane (Type 2).	[48,51,52]	
chitin—beetles The chitin fibrils embedded in a protein matrix are uniaxial with refractive indices of *n*_o_ = 1.70 and *n*_e_ = 1.54	[2,28,53,54]	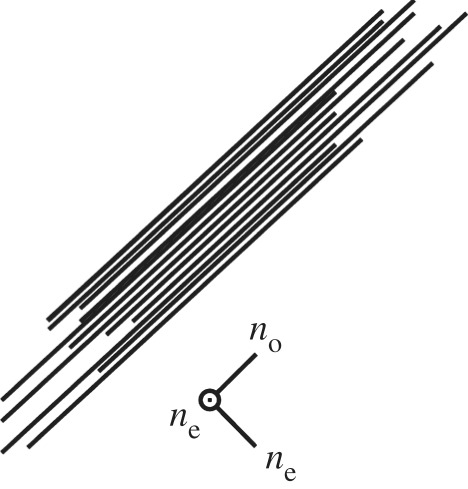
*intrinsic anisotropy*—chirality		
chitin—beetles	[2,25,28,55–57]	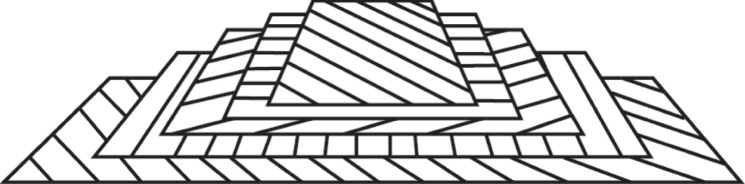
chitin—crustaceans Helical progression of chitin layers found in arthropod cuticles—an oblique cut demonstrates the nested arcs / Bouligand planes	[58,59]	
chitin—butterfly In many lepidopteron scales Chitin can also form a variety of minimum energy surfaces such as single gyroids.	[60–63]	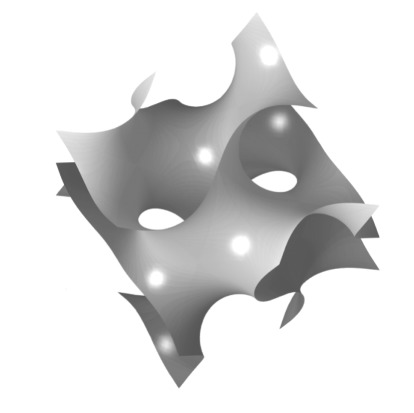
*form birefringence* Form anisotropy occurs when the length scale of individual components is less than the wavelength of the light but the overall size is much greater than the wavelength.		
chitin—butterflies	[64]	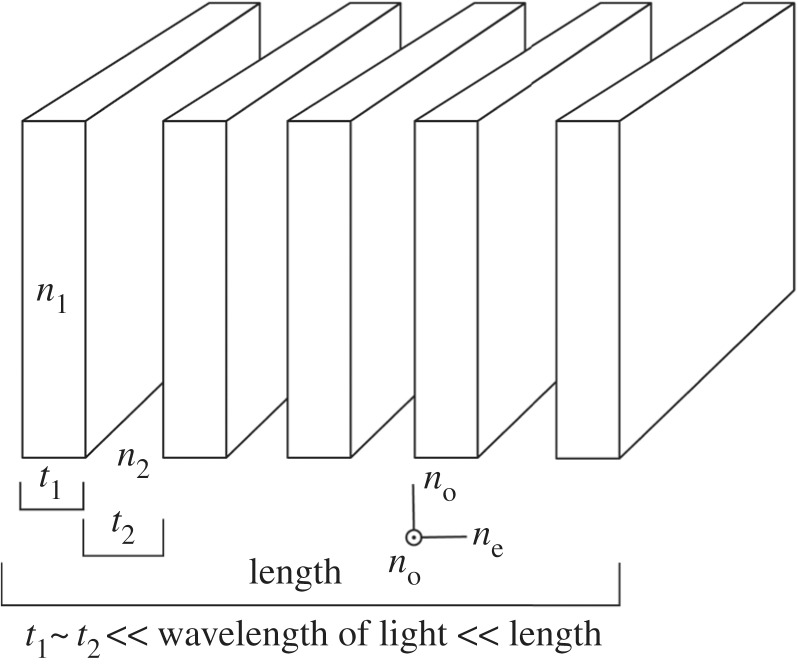
chitin—Orthopteran The assembly behaves as a positive uniaxial crystal where the optic axis is perpendicular to the plane of the plates.	[65]	
*Structural anisotropy* Structural anisotropy occurs when the length scale of anisotropy is comparable to the wavelength of light. At this scale, the polarization of reflected light is controlled by asymmetric scattering and interference/diffraction (rather than anisotropy in the refractive index).		
anisotropic vesicles—stomatopods Hollow ovoid vesicles with aspect ratios of 2–3 found in the maxilliped cuticle	[44]	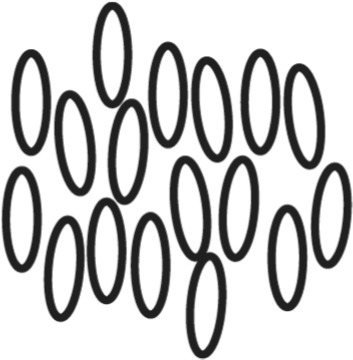
diffraction gratings—insects Diffraction grating is periodic in the x-direction.	[2,4,66]	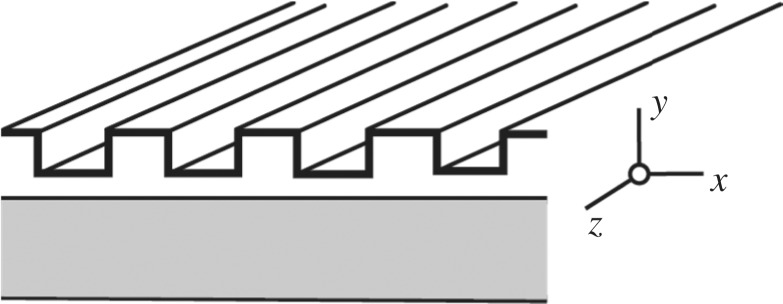

## 3. Chitin in golden beetles: increasing the reflectivity via polarization mode conversion

**Figure 1.**
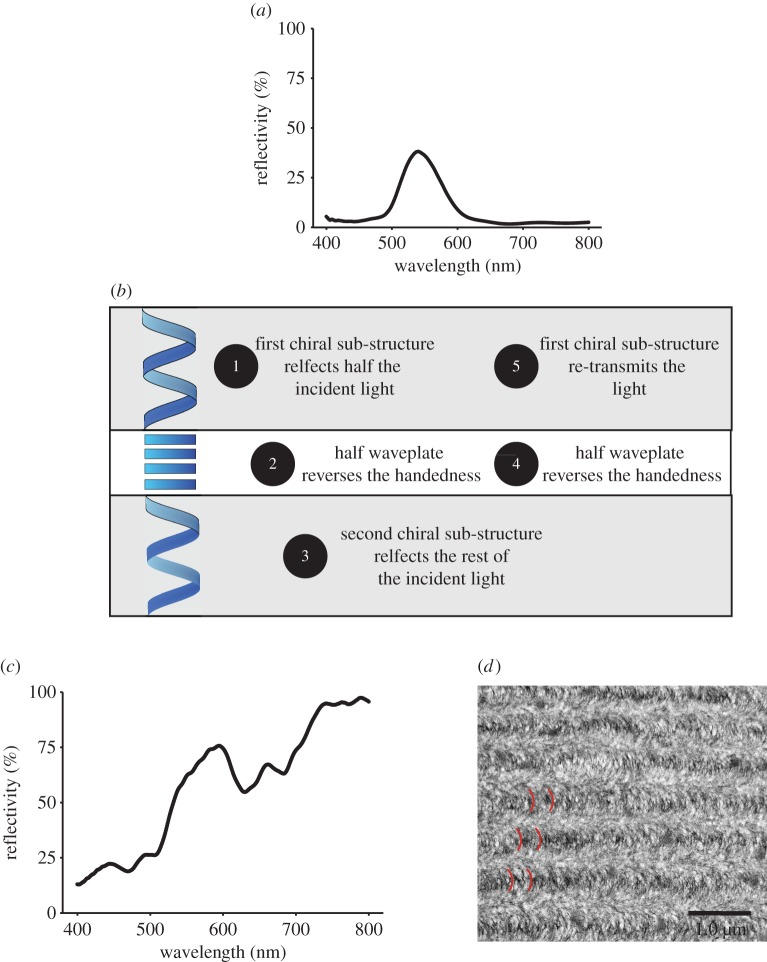
Visible reflection spectra from two species of beetle. (*a*) *Chrysochroa aurora*: reflection spectrum illustrating the green coloration of the beetle. (*b*–*d*) *Chrysina resplendens*: (*b*) diagram of the three sub-structures, two helical layers of the same handedness and one half-wave retardation plate. The combination effect is a mechanism that creates the greater than 50% reflectivity. (*c*) Reflection spectrum showing the broadband gold coloration and that the reflectivity is greater than 50% in the yellow region of the spectrum. Note the further increase reflectivity in the near infrared. (*d*) An oblique TEM section of first set of layers in *C. resplendens* illustrating the nested arcs that characterize the classic Bouligand planes that reveal the helical structure.

The chiral structures found in beetles are documented in many publications and the optics of the cholesteric or chiral nematic-like chitin is well understood [2,7,28]. A fact that is not often discussed is that this mechanism imposes a limit on the maximum reflectivity of material. For incident unpolarized light, 50% reflectivity is a fundamental maximum. Resolving the incident light into equal amounts of left-handed and right-handed circularly polarized light, it can be seen that the component that matches the handedness of the structure will be transmitted and the component of the light that has the opposite handedness will be reflected. In reality, a degree of disorder and defects in the structures make this a theoretical maximum and the reflectivity is always somewhat lower. Figure 1*a* illustrates this in the iridescent green species *Chrysochroa aurora*, where with one set of chiral layers, the spectral reflection of incident unpolarized light reaches a maximum reflectivity of approximately 40% at 540 nm.

Having said that, this maximum 50% reflectivity threshold is not true for all beetles. Michelson reported one of the first examples of structural coloration in 1911 for the species *Chrysina resplendens* (previously *Plusiotis resplendens*) [24]. This is a beetle with a remarkable highly reflective gold appearance whose optical structure has elegantly evolved a way to bypass the 50% reflectivity limit (figure 1*b*). The broadband reflection (figure 1*c*) is created by a progression in the pitch of the helices. This is shown in figure 1*d* with the nested arc formations seen in each the layers. First characterized by Bouligand in 1965 [67], and known as Bouligand planes, each arc spans one half of the chiral pitch. However, the structure in this species is not just comprised of one sub-structure, but three (figure 1*b*) [53]. The first chiral sub-set of layers reflects half of the incident light as left-handed polarized and transmits the other half as right-handed. There then exists a birefringent uniaxial (non-chiral) layer that acts as a half-wave plate converting the right-handed polarization into left-handed [53]. The light transmitted through the wave plate is then completely reflected from a third chiral structure, another helical architecture of the same handedness as the first sub-set of layers. The light now returning through the structure is then converted again from left- to right-handed by the half-wave plate and transmitted as left-handed polarized light back through the first. Such a structure with two helical layers surrounding a half-wave plate has an ideal limit of being 100% reflective across the spectral reflection bands. Figure 1*c* shows this is indeed the result, with the gold appearance being approximately 70–80% reflective and the reflectivity increasing towards 100% in the near infrared. Again while defects and the wavelength dependence of the retardation plate keep the reflectivity below the absolute 100% maximum, it is still appreciably greater than the single layer maximum of 50%. This is an elegant example of how the intrinsic polarization properties of animal optical materials, and their arrangement within the structure, have been used to manipulate the light and create a mechanism of increased reflectivity.

## 4. Guanine in silvery fish: increasing the angular reflectivity via birefringence

The example of *C. resplendens* illustrates how intrinsic polarization properties such as chirality and birefringence can influence the reflectivity of a structure; however, very little experimental evidence exists for the ultimate causation. Studies of highly reflective structural colours in the pelagic environment provide a more complete narrative, in terms of how both the unique optical adaptations and light environment combine for improved camouflage.

Reflections from many pelagic mid-water fish, such as Atlantic herring, *Clupea harengus,* are produced by a multilayer ‘stack’ of guanine crystals with cytoplasm gaps [30]. Many fish have two separate forms of these stacks: (i) those in the *stratum argenteum* (a subdermal layer of the skin) and (ii) those that lie on the inner surface of the scales [9,30]. Within the skin the broadband reflectivity is created by a broad range of cytoplasm spacings between the guanine crystals, although there is also somewhat of a distribution in the thicknesses of crystals themselves [1,34,36,48]. The optical effects are theoretically underpinned by the physics of the localization of light [35,36,68]. While this is a typical picture of what is known as a distributed Bragg reflector, the intrinsic polarization properties of birefringent guanine create a very unusual type of reflector that affects the silvery camouflage in a remarkable way.

It has been known since the 1960s that biogenic guanine crystals, which are a mixture of pure guanine and hypoxanthine, are biaxial and highly birefringent with refractive indices of approximately 1.85, 1.81 and 1.46 along the different morphological axes of the crystal [49]. The standard morphological form of guanine, which here we term Type 1, is present in various fish reflectors described in [1,49,50] and spiders [1,48,51]. Type 1 crystals have both the larger refractive indices in the broad plane of the crystal, and thus parallel to the stacking direction. The atomic basis of this intrinsic anisotropy, is described in Levy-Lior [50], and relates to the stacking structure of the H-bonded guanine molecules, which has direction-dependent molecular polarizability, and thus refractive index. It was also demonstrated by Levy-Lior in 2008 [50] that the orientation of the crystal optical axes (i.e. the orientation of the refractive indices relative to the broad plane of the crystal/stacking direction), differs from the lowest energy configuration. Since, for normal incidence reflection, the observed orientation acts to maximize the refractive index contrast across interfaces, it was suggested that the crystal morphology might arise through biological control mechanisms.

**Figure 2.**
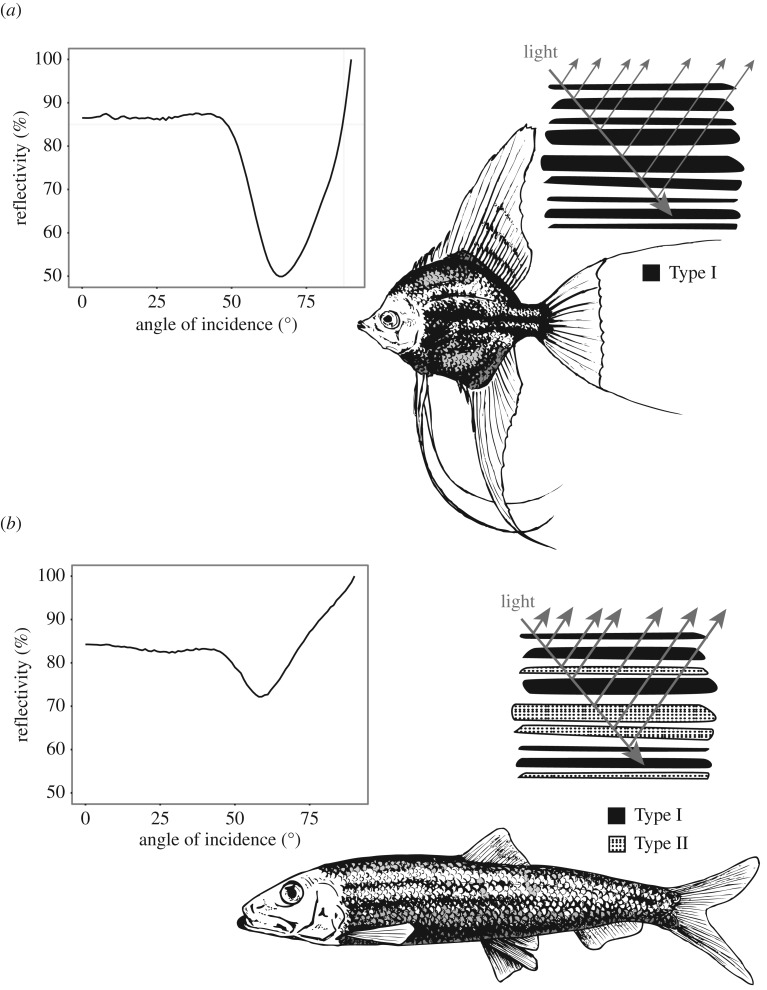
Reflectivity as a function of viewing angle from the sides of silvery fish. *Pterophyllum scalare* (*a*) inset illustrates the distributed Bragg reflector of one optical type of guanine. The single-Type structure results in the decrease of the reflectivity to 50% at Brewster's angle. *Clupea harengus* (*b*) inset illustrates the 2-Type reflector and how this now maximizes the reflectivity over all viewing angles. The plot uses the transfer matrix simulation procedure described in Jordan *et al*. [37].

Figure 2*a* illustrates an example reflectivity spectrum from a guanine-cytoplasm reflector containing the standard form of Type 1 guanine crystal, which approximates the crystals as uniaxial with refractive indices 1.83 and 1.46. As a function of viewing angle, it can be seen that the reflectivity decreases to 50% at Brewster's angle (around 67 degrees) due to polarization properties of the layer interfaces. Clearly, for a predator viewing a silvery fish at such an angle there would a substantial intensity contrast of the prey item against the background. ‘Non-polarizing reflectivity’ over all angles of incidence, however, creates the optimal 100% intensity-matching camouflage to occur over all viewing angles [6]. The reflectors found in *C. harengus* display a set of polarization properties that go some way to matching this ideal, employing a structure without a defined *overall* Brewster's angle, which enables reflectivity greater than 50% for all viewing angles (figure 2*b*). In principle the mechanism does enable 100% reflectivity for the visible regime [6,36]. Recent studies have shown the *stratum argenteum* of *C. harengus* contain two optically distinct populations of guanine crystals, a second population that we have termed Type 2 crystals [6]. Though morphologically similar, in these crystals the larger refractive index is in the direction normal to the plan of the crystal surface.

The consequence of the different orientation of the refractive indices for the two crystal Types is a that a wide angular separation exists between the Brewster angles of each interface of the crystals in the stack (ranging from approx. 33–67 degrees). The basic intuition as to how this enables high reflectivity for all viewing angles is that because the reflection minima for each interface for the tangential polarization component are at different angular positions, the structure as a whole is able to reflect all polarizations of light at all angles of incidence [6]. A more rigorous description of this mechanism is described by Jordan *et al*. [35,36] in terms of Anderson localization and the structural property of the localization length. These works demonstrate that the thickness disorder is sufficient to produce an angularly insensitive broadband reflection over the visible regime. A stack structure containing purely Type 2 crystals can also produce polarization-insensitive reflection [6]. These hypothetical structures are, however, less efficient at producing high reflectivity over all viewing angles, and our simulations indicate that the *stratum argenteum* of *C. harengus* approaches the optimal ratio of Type 1 to Type 2 to produce the highest reflectivity over all viewing angles. By producing near polarization-neutrality for reflections over all angles of incidence, this two-Type crystal system in pelagic fish ensures a greater total reflected intensity that more closely matches the open-water background light field than equivalent reflectors consisting of a single crystal Type, or constructed from isotropic materials.

## 5. Discussion

In general, we still understand very little of the complete picture of animal structural coloration, from optical mechanisms through to the behavioural ecology. In many common animals the structural mechanisms are yet to be investigated or explained. For example, a number of species of UK grass moths exhibit a remarkable gold coloration. The greatest gap in our knowledge centres on the genetic mechanisms that control the components of self-assembly and coordination of both the structures as a whole and the intrinsic optical properties of the biological materials. However, this paper provides evidence for a new concept in how we think about the evolution of these materials: the underlying message in this work is that the intrinsic polarization properties of biological reflectors, such as birefringence, are under selection. Exploiting a range of distinct, symmetry-breaking optical mechanisms that are accessible through the use of anisotropy and chirality, animals have evolved optical solutions to increase reflectivity particularly in the context of camouflage. With a limited sub-set of biological materials, adapting the intrinsic polarization properties within an optical structure permits simple access to a greater regime of optical responses. Put simply, the selection of specific anisotropic properties in one-dimension structures is a simpler way of creating optical responses than moving to more complicated three-dimensional systems. For example, stomatopods have a pre-existing sensory bias of horizontally polarized light, which has directed a change from an isotropic reflective structure to an anisotropic ordered one, a structure that introduces a horizontally polarized dimension to a visual signal [69].

It is always important to connect what we learn from animal optical structures with developing improved optical technologies. Interestingly, there are several synthetic structures that parallel the way the polarization properties of these organic materials affect the optical response. The fish reflectors discussed in Jordan *et al*. [6] were originally described in relation to a device called an omnidirectional reflector [70], although guanine crystal stacks themselves do not fulfil the criteria to be termed an omnidirectional reflector. Here we discuss another parallel that can be made. The birefringence of the guanine crystals and inter-layer refractive index contrast of the guanine crystals with the cytoplasm gaps are within the ‘giant birefringent optics’ (GBO) regime described for engineered multilayer reflectors made from birefringent polymers described by Weber *et al*. [71]. Multilayer reflectors constructed using GBO design criteria exploit a generalization to Brewster's law of polarization for birefringent materials [72], whereby the Brewster angle can be engineered to a desired angle via the orientation and magnitude of the birefringence. In principle, these GBO criteria enable multilayer structures to have a Brewster angle anywhere between 0 and 90 degrees, which offers enhanced control of polarization by reflection over isotropic reflectors. However, it is worth highlighting some key differences that distinguish the fish reflectors from standard GBO devices. Firstly, there are three different classes of layer present in the fish (the isotropic gaps and the two types of crystal), whereas there are generally only two types of layer present in man-made GBO designs. The result of this is that the degree of polarization maxima in the fish multilayers does not relate directly to the Brewster relations for each interface (see fig. 1 in Jordan *et al*. [6]), and a statistical averaging of the reflection across the interfaces in the stack layers is present [35]. Secondly, there is a greater variance in the thicknesses of the layers in the fish multilayer structure than in most GBO designs that are periodic or have systematically varying thicknesses. Thirdly, spatial/ensemble averaging of the optical properties of the fish multilayer occurs, which acts to improve uniformity, whereas GBO designs refer to a single stack realization.

The distributed birefringent Bragg reflectors in pelagic fish highlight a further important aspect of an animal's visual ecology and the evolution of animal structural optics. In order to make claims about the effectiveness of cryptic camouflage in different dimensions of light, such as intensity or polarization, direct evidence is required for increasing the animal's survivability. For example, Cuthill [73] was able to prove through survivability analysis that disruptive coloration acts as a form of camouflage. To date, no experimental evidence exists for whether matching the polarization of the background in an underwater light environment makes any difference to survivability. Moreover, this leads to a more general and key final point. In cases of camouflage, natural selection acts on structural coloration depending only on a receiver/predator's visual system. Human experience, being limited to a trichromatic colour system, and with limited vocabulary describing only our own visual perception, lacks the ability to explain perceptual appearance for animals with very different colour vision. The same is true of polarization, but seems often forgotten. The inter-relationship between the reflectivity and the polarization properties must always take into account the receiver's visual system; animals know nothing of angle or degree of polarization or the mathematical constructs we use to describe them.

## 6. Conclusion

In this study we focused upon the role the intrinsic polarization properties of biological materials, such as the birefringence of guanine and the chirality of chitin, play in controlling animal structural coloration. The golden reflectors in the beetle *C. resplendens* and the silvery reflectors in the fish *C. harengus* both illustrate that the intrinsic polarization properties can act to control and improve the overall percentage reflectivity of the structure. Thus, these intrinsic polarization properties directly influence the intensity component of visual information. The fish reflector in the pelagic environment provides a model example for such an adaptation of optical properties, where improved reflectivity as a result of intrinsic polarization properties acts to improve the selective advantage of the reflector. Future studies of biological reflectors that include anisotropic materials should always address whether the polarization properties may be an adaptation to enhance reflectivity, and should also consider carefully the context of intended receivers' visual system.
